# Polylactide Nanoparticles as a Biodegradable Vaccine Adjuvant: A Study on Safety, Protective Immunity and Efficacy against Human Leishmaniasis Caused by Leishmania Major

**DOI:** 10.3390/molecules27248677

**Published:** 2022-12-08

**Authors:** Sana Ayari-Riabi, Noureddine Ben khalaf, Balkiss Bouhaouala-Zahar, Bernard Verrier, Thomas Trimaille, Zakaria Benlasfar, Mehdi Chenik, Mohamed Elayeb

**Affiliations:** 1 NanoBioMedika Team, Laboratoire des Biomolécules, Venins, et Applications Théranostiques (LBVAT) LR20IPT01, Institut Pasteur Tunis, Université Tunis El Manar, BP 74, 13 Place Pasteur, Tunis 1002, Tunisia; 2 Life Sciences Department, College of Graduate Studies, Arabian Gulf University, Manama P.O. Box 26671, Bahrain; 3 Laboratory of Immunopathology, Vaccinology and Molecular Genetics, Institut Pasteur de Tunis, BP 74, 13 Place Pasteur, Tunis 1002, Tunisia; 4 Laboratoire de Biologie Tissulaire et d’Ingénierie Thérapeutique, Univ Lyon, CNRS, Université Claude Bernard Lyon 1, UMR 5305, 7 Passage du Vercors, CEDEX 07, 69367 Lyon, France; 5 Ingénierie des Matériaux Polymères, Univ Lyon, CNRS, Université Claude Bernard Lyon 1, INSA Lyon, Université Jean Monnet, UMR 5223, CEDEX, 69622 Villeurbanne, France; 6 Service des Unités Animalières, Institut Pasteur Tunis, BP 74, 13 Place Pasteur, Tunis 1002, Tunisia

**Keywords:** PLA nanoparticles, *Leishmania major*, recombinant histone H2B, vaccine adjuvant, parasite’ challenge, lesion swelling

## Abstract

Leishmaniasis is the 3rd most challenging vector-borne disease after malaria and lymphatic filariasis. Currently, no vaccine candidate is approved or marketed against leishmaniasis due to difficulties in eliciting broad immune responses when using sub-unit vaccines. The aim of this work was the design of a particulate sub-unit vaccine for vaccination against leishmaniasis. The poly (D,L-lactide) nanoparticles (PLA-NPs) were developed in order to efficiently adsorb a recombinant *L. major* histone H2B *(L. major* H2B) and to boost its immunogenicity. Firstly, a study was focused on the production of well-formed nanoparticles by the nanoprecipitation method without using a surfactant and on the antigen adsorption process under mild conditions. The set-up preparation method permitted to obtain H2B-adsorbed nanoparticles H2B/PLA (adsorption capacity of about 2.8% (*w*/*w*)) with a narrow size distribution (287 nm) and a positive zeta potential (30.9 mV). Secondly, an in vitro release assay performed at 37 °C, pH 7.4, showed a continuous release of the adsorbed H2B for almost 21 days (30%) from day 7. The immune response of H2B/PLA was investigated and compared to H2B + CpG7909 as a standard adjuvant. The humoral response intensity (IgG) was substantially similar between both formulations. Interestingly, when challenged with the standard parasite strain (GLC94) isolated from a human lesion of cutaneous leishmaniasis, mice showed a significant reduction in footpad swelling compared to unvaccinated ones, and no deaths occurred until week 17th. Taken together, these results demonstrate that PLA-NPs represent a stable, cost-effective delivery system adjuvant for use in vaccination against leishmaniasis.

## 1. Introduction

Leishmaniasis, a neglected tropical disease, is the third most challenging vector-borne disease after malaria and lymphatic filariasis. According to the World Health Organization’s (WHO) published data, it has been reported that leishmaniasis is endemic to approximately 100 countries of the world [1]. Cutaneous leishmaniasis (CL) is the most common form of leishmaniasis. The annual registered cases are around 0.7–1.2 million across the globe. The disease is caused by the protozoan *Leishmania* parasite [2]. The principal vector responsible for mammal-to-human transmission is the Phlebotomine sandfly. Two distinct morphological forms of *Leishmania* have been identified in its life cycle: the promastigote is present in the vector, and the amastigote moves into the monocytes/macrophages of the human or mammalian host [3]. CL is a skin infection that reduces patients’ quality of life and imparts psychological problems and social stigmatism [4].

When BALB/c mice, an inbred strain of mice, are infected with the *L. major* parasite, the maturation of a Th2 immune response is triggered by the interleukin-4 (IL-4) produced during the first two days. This IL-4 rapidly renders parasite-specific CD4^+^ T cell precursors unresponsive to interleukin-12 (IL-12) [5].

Effective regulation requires macrophage activation and nitric oxide (NO) in response to the Th1-produced cytokine IFN-γ. Disease prognosis can be improved by overcoming problems such as low efficacy, systemic toxicity, insufficient drug within macrophages, poor antigen presentation to cells and expensive care. Vaccination may be the most appropriate strategy in this context.

Adjuvants for *Leishmania* vaccines to date are categorized into two types: (i) immunostimulatory molecules and (ii) nanoparticulate and/or delivery systems. The first ones include Bacille Calmette–Guerin (BCG) emulsified with antigens, e.g., *Leishmania amazonenzis* isolate IFLA/BR/1967/pH 8 [6], autoclaved *Leishmania major* ALM or GP63 [7,8], the monophosphoryl lipid (MPL) with LEISH-F1 or LEISH-F2 peptides [9,10], the interleukin-12 (IL-12) with pSP Leish-tec peptide [11], saponin with A2 Leish-tec [12], GLA with SMT Leish-tec [13] and CpG-ODN with KMP-11 Leish-tec [14]. However, immunostimulatory adjuvants suffer from rapid clearance and safety issues [15]. Nano-based delivery systems (NDS) applied as potential adjuvants in anti-leishmanial vaccines may be a useful alternative to conventional bacterial adjuvants and virus vectors [16]. An NDS could potentially deliver target vaccines to the site of action within the host’s body, enhance immune reactions by facilitating antigens’ absorption and uptake by antigen-presenting cells (APCs) [17], prevent its degradation such as peptides, proteins, or oligonucleotides [18], promote their controlled release and modulate the type of immune responses [19,20]. The particulate adjuvants, for example, liposomes, polymeric microspheres, and emulsions, have been utilized effectively to deliver *Leishmania* antigens in preclinical models of leishmaniasis, as well as other infectious diseases [21]. Liposomes (LPS) and derivatives have been widely studied. Despite the relevance of LPS, some shortcomings are also associated with these lipid-based nanocarriers, such as leakage of the entrapped moiety, oxidation, hydrolysis, and inadequate stability [22]. The polymeric particles, when used as adjuvants, could develop more potent immunogenicity against *Leishmania* antigens [23]. Several polymers, such as polylactide (PLA), polyglycolide (PGA), poly-lactide-co-glycolide (PLGA), poly-caprolactone (PCL), poly-cyanoacrylate (PCA) and natural protein polymers, such as albumin and gelatin, and polysaccharides, have been investigated as vaccine carriers. The polymer that has been explored most extensively is polylactide (PLA). The FDA has approved the use of this compound in human applications due to its biocompatibility and lack of toxicity [23,24]. Currently, PLA is commercialized as part of several nanomedicine tools. For vaccine purpose, antigens formulated with PLA-NPs induce broad and potent humoral and cellular immunity in mice, rabbit and macaque models [25,26]. Other researchers have shown that a mycobacterial antigen adsorbed on lamellar particles of lactide polymers have induced cellular immunity [27].

Another strategy to control leishmaniasis is antigen-target-specific vaccines. These are categorized into three types: (i) live *Leishmania* parasites; (ii) killed *Leishmania* or parasite fractions (first generation), and (iii) *Leishmania* recombinant proteins (second generation) or DNA molecules (third generation) [16]. DNA vaccines are of particular interest because they can effectively induce both CD8+ and CD4+ T cells and produce long-lived antigens and properly folded polypeptides [28]. However, these are still in the early phases of clinical trials. Some trials of developing first-generation vaccines have failed to provide convincing results in phase III due to standardization and safety issues, whereas others are still in earlier phases. Second-generation vaccines, e.g., leish-111f, suffer from a lack of an appropriate adjuvant [9,21]. Among them, the histone H2B protein was described as a potential candidate [7,10]. The histone protein H2B forms with H2A, H3 and H4, the major constituents of the nucleosome in the nucleus of eukaryotic cells [29]. This protein is conserved among various species of *Leishmania*, i.e., *L. major*, *L. infantum*, *L. donovani* and *L. tropica*. Previous studies investigated the immunogenicity and the protective role of the recombinant H2B protein from *L. major* [30]. H2B, in combination with CpG-ODN, confers effective protection to sensitive BALB/c mice infected with the virulent strain of the *L. major* parasite [31]. Later, Meddeb-Garnaoui et al. showed that this recombinant protein H2B induced a specific Th1-type cellular response in individuals who recovered from cutaneous leishmaniasis infection [32].

The purpose of this research was, on the one hand, to develop a particulate vaccine system based on PLA, a polymer that has received FDA approval, and on the other hand, to protect mice against *L. major* parasite infection. To do this, nanoparticle dispersion was developed using PLA polymer (PLA-NPs). Then, BL21 *Escherichia coli* strain cells were used to express the histone H2B protein, a sub-unit vaccine candidate for leishmaniasis. When animals were experimentally infected with the parasite *L. major* promastigotes, the immune protection of immunized mice was investigated and compared to CpG-ODN, a TLR agonist compound.

## 2. Results

### 2.1. Characterization of PLA NPs

**Figure 1:**
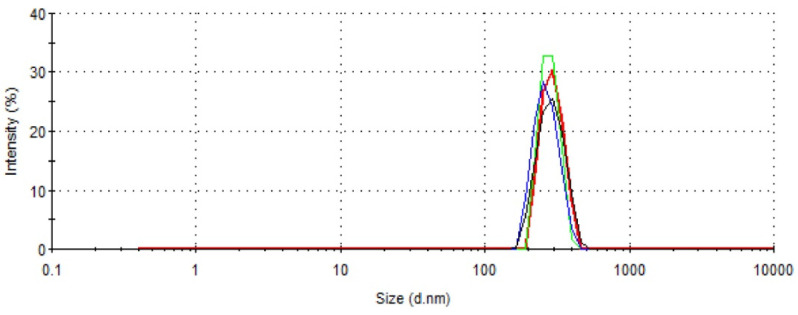
Size distribution of PLA-NPs using DLS technique. NPs were prepared by a nanoprecipitation process in one single step without using surfactant.

The nanoparticles were obtained by the nanoprecipitation method without adding surfactant. Optimum experimental conditions were as follows: 0.2 g PLA polymer was dissolved in acetone (10 mL) and added to MilliQ water (1v/3v). A white and milky suspension was obtained after the formation of the nanoparticles. The production yield, based on recovered PLA after solvent evaporation, was in the 70−80% range. According to the DLS results, the obtained nanoparticles had a mean hydrodynamic diameter of 287.4 nm (±10); and a polydispersity index (PDI) of 0.14 (±0.06) (Figure 1). This indicates that NP distribution was in a quite narrow range. Zeta potential is one of the important parameters affecting the stability of nanoparticles. Their zeta potential value was −45 mV (±5), prone to ensure colloidal stability through electrostatic repulsion.

For stability analysis, nanoparticles (4 mg/mL) were kept at +4 °C for several months. No significant variation in particle size was observed until month 18.

### 2.2. Characterization of a Recombinant H2B Protein

**Figure 2:**
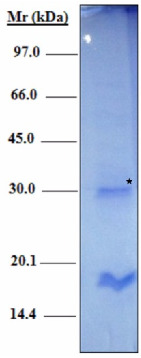
SDS analysis of *L. major* recombinant H2B. Protein was expressed in BL21 *Escherichia coli,* then purified by affinity chromatography over Ni-NTA resin and analyzed using SDS-PAGE (15%) followed by Coomassie blue staining. (*) indicates an additional band that corresponds to H2B dimeric form. Mr, Molecular weight markers (kDa) (GE Healthcare).

Recombinant protein H2B was produced in *Escherichia coli* BL21 bacteria (*E. coli* BL21) using the pET prokaryotic expression system. Proteins were then purified by affinity chromatography over Ni-NTA resin, and purity was assessed by SDS-polyacrylamide gel electrophoresis. Staining of the gel with Coomassie blue revealed two bands of 16 kDa and 30 kDa, respectively, Figure 2. As previously described based on Western blot analysis, bands of 16 kDa and 30 kDa correspond to recombinant H2B monomeric and dimeric forms, respectively [31].

### 2.3. Adsorption Efficiency and Zeta Potential of H2B/PLA Particles

**Figure 3:**
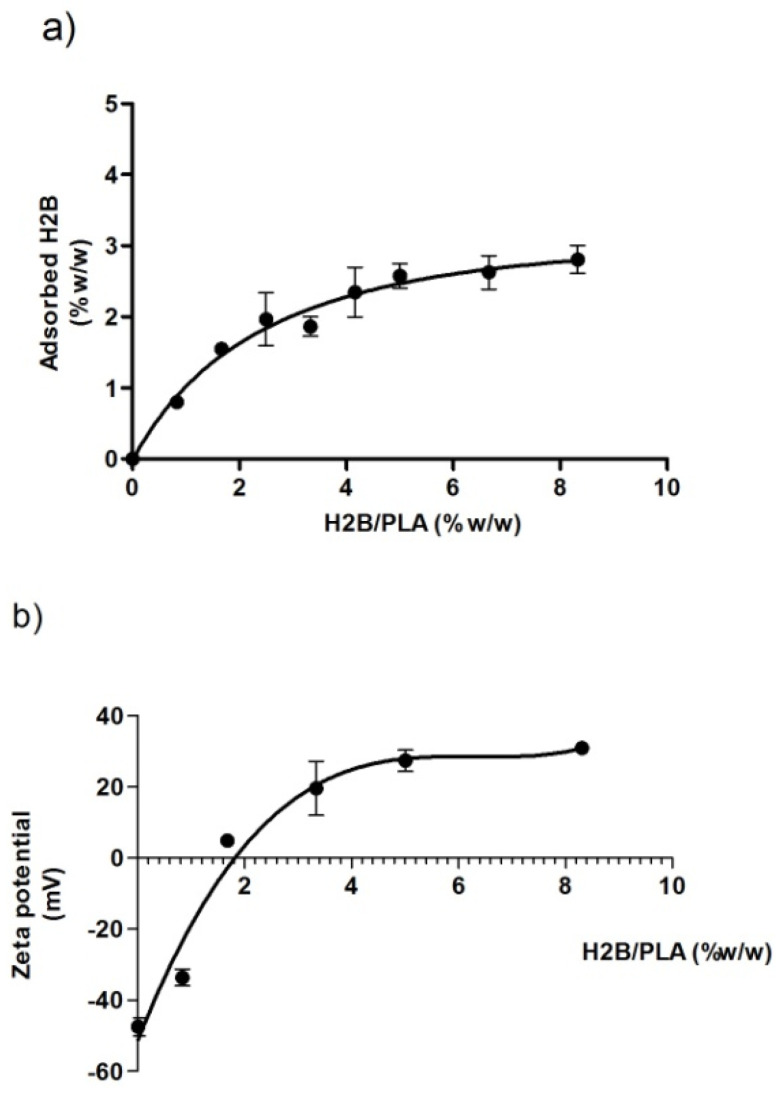
Adsorption curves of H2B/PLA-NPs. (**a**) Adsorption efficiency of the peptide/particles (0.06% *w*/*v*) at different protein-to-particle ratios. (**b**) Zeta potentials of H2B/PLA as a function of initial protein amount (% *w*/*w*).

The characteristics of H2B-adsorbed PLA nanoparticles are reported in Figure 3. The NP concentration was fixed at 0.06 w% (i.e., 0.6 mg/mL). The adsorption process was followed by monitoring both the amount of adsorbed protein and the surface charge of H2B/PLA. The maximum adsorption was reached when an introduced protein/particle ratio ranged from 5 to 8.3 w%. Thus, the adsorption capacity was 2.8% *w*/*w* of protein particles (Figure 3a). Zeta potentials were followed as a function of introduced protein amounts under the adsorption process, as its strongly cationic character is expected to induce charge inversion (Figure 3b). Flocculation of the colloid was observed around the neutralization point (~2 w% protein/particle ratio). The colloidal stability was restored at an introduced H2B/particle ranging from 5 to 8.3% with a zeta potential of +30 mV and no longer changes, indicating the saturation of the colloid surface.

In further studies, H2B-coated NPs were prepared in the presence of excess protein to avoid the flocculation process (5 w% initial protein/NP ratio) and washed from unbound protein (centrifugation/redispersion steps). The H2B/PLA exhibited a size and a zeta potential of 341 nm and +30.9 mV, respectively, Table 1.

### 2.4. In Vitro Desorption of Adsorbed H2B

**Figure 4:**
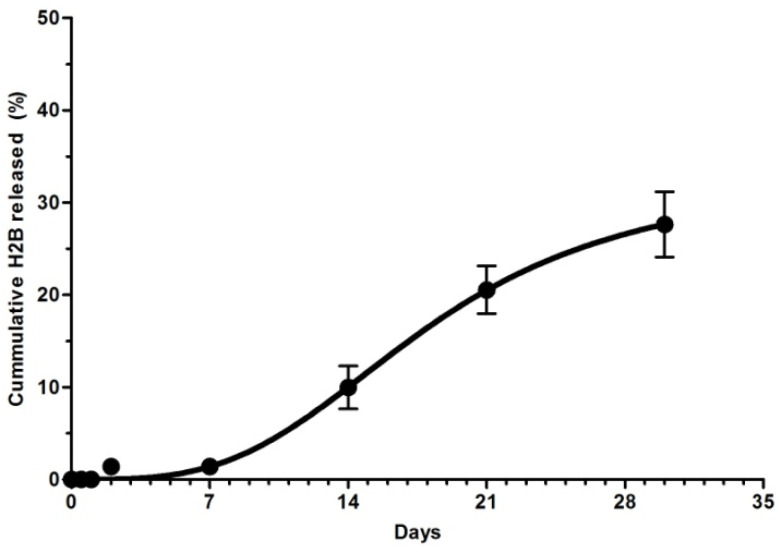
Release of adsorbed H2B peptide over time. A known amount of H2B/PLA was suspended in PBS buffer solution (pH~7.4) (GIBCO) at 37 °C. Protein desorption was followed at predetermined time intervals. Supernatants were collected at each point, and protein concentration was determined using a micro BCA assay kit (St. Louis, MO, USA, Sigma-Aldrich). Desorption rate was expressed as the cumulative percentage.

The H2B/PLA-NPs were incubated in PBS buffer (pH 7.4) at 37 °C. The amount of protein in each supernatant was quantified at a fixed time. As shown in Figure 4, protein release followed a specific profile over time. No protein was detected in the supernatant collected during the first three days until day 7. Continuous H2B release was quantified from day 7 to day 21 with a cumulative percentage value of 20%. After that, the release continues to slow down until the end of the test and the maximum percentage value of 30% was obtained (Figure 4).

### 2.5. Assessment of the Antibody Response

**Figure 5:**
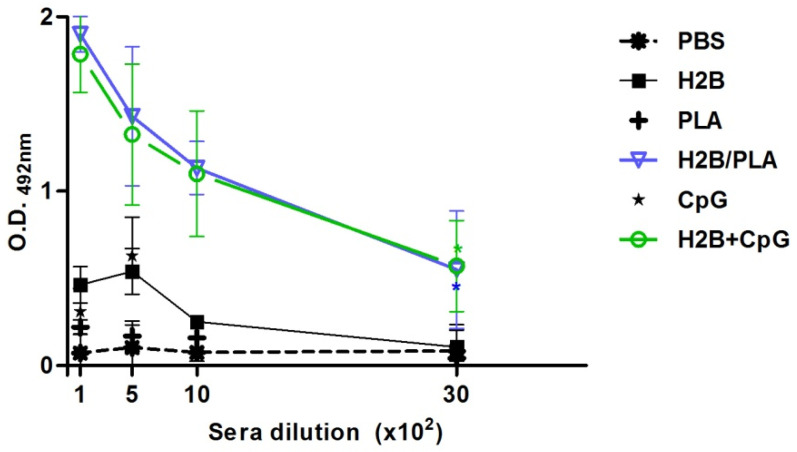
Specific anti-H2B antibody immune response. Pooled sera from each group were reacted in ELISA with recombinant H2B protein. Total IgG titers were calculated for each group. Titers were scored positively at the last dilution of immune sera. Results are represented as mean ± standard deviation of optical density (O.D.). (*) *p* < 0.05 between PBS control and protein-received groups.

To demonstrate an antigen-specific adjuvant effect, BALB/c mice were subcutaneously immunized two times on days 1 and 14, with the following vaccine formulations; H2B alone and H2B/PLA-NPs. As a positive control, H2B plus CpG-ODN7909 was also included because it triggers cytotoxic immunity. For non-specific responses, one group was given PLA-NPs, while the other was given CpG-ODN7909 only. For infection control, one group was given PBS alone. Specific IgG titers were assessed by ELISA on day 30 at the end of the immunization schedule. A significant increase in the specific IgG titer was found after one month of primary immunization for groups vaccinated with either H2B/PLA-NPs or H2B + CpG in contrast to groups that were exposed to soluble protein (Figure 5). There was an increase in the average antibody levels. These results indicate that PLA-based vaccine formulation induced humoral immunity.

### 2.6. Analysis of the Anti-H2B IgG Isotype

**Figure 6:**
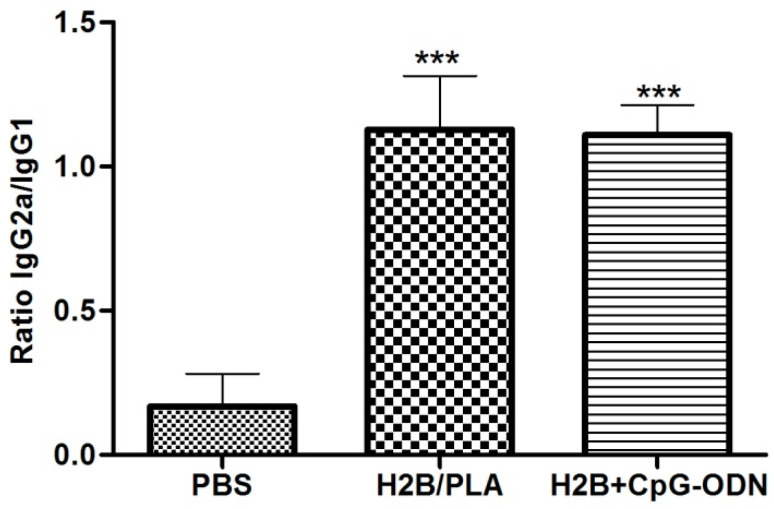
IgG isotype ratio in pooled sera from immunized mice. Results are represented as mean ± standard deviation (SD) of optical density (O.D.) values obtained from 5 mice per group. (***) *p* < 0.001 between PBS control and protein-received groups.

IgG1 and IgG2 antibody isotypes are markers of the humoral and cellular immune response, respectively. The evaluation of their titers allows them to determine by which pathway, Th2 or Th1, the immune system reacts to H2B/PLA formulation. Both adjuvants (particulate, PLA and molecular, CpG) produced a significant IgG2 isotype level. This suggested that the PLA immune response tends towards a Th1-type response, as confirmed with the CpG-ODN adjuvant (as control) (Figure 6).

### 2.7. Protective Potency of H2B/PLA in Mice

The protective potential of antigen adsorbed onto PLA was compared with that of CpG-ODN7909, which has previously been shown to be a Th1 response enhancer.

**Figure 7:**
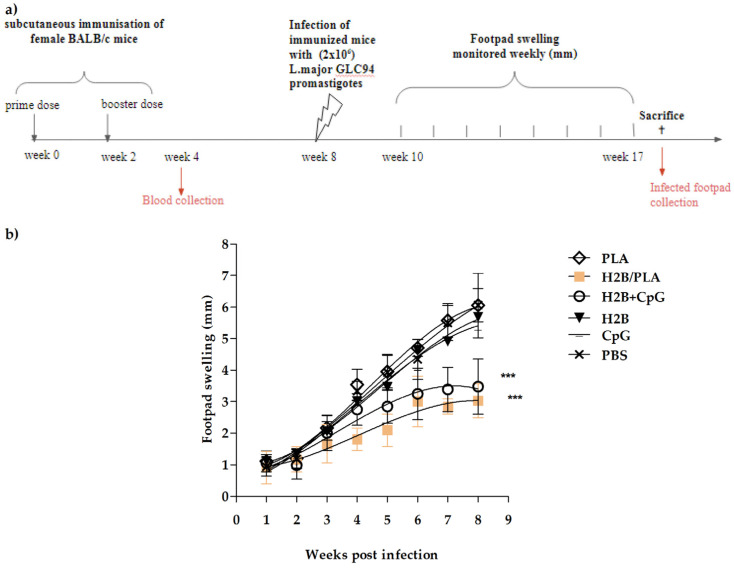
Immune protection of vaccinated BALB/c mice against *Leishmania major* challenge. (**a**) The timeline of the in vivo experiment. Female BALB/c mice (6–8 weeks old) were vaccinated twice on day 1 and day 14 with H2B/PLA (25 µg/protein), H2B (25 µg) + CpG or soluble H2B (25 µg). The control groups received PBS, PLANPs or CpG. Mice were infected with (2 × 10^6^) *L. major* GLC94 promastigotes by subcutaneous injection on the right footpad. (**b**) Footpad swelling. Lesion sizes were monitored weekly for up to eight weeks. The mean lesion size ± SD is shown (*n* = 8). (***) *p* < 0.001 between PBS control and protein-received groups.

The challenge tests were performed using the standard parasite strain, MHOM/TN/94/GLC94 (GLC94), isolated from a human lesion of cutaneous leishmaniasis (CL). This isolate belongs to the species of *L. major*, zymodeme MON25 and is the most virulent strain [31,33]. A high dose (2 × 10^6^) of GLC94 parasites was inoculated through subcutaneous injection into the right footpad six weeks after the booster dose (week 8). Then, the course of the infection was recorded weekly for eight weeks (weeks 10 to 17) Figure 7a. The progression of lesions was similar in all groups of mice during the first three weeks post infection. Among the groups that received H2B antigen, PLA NP gave the best results, followed by CpG-ODN, applied as a control adjuvant. Since the soluble protein itself is immunogenic, a moderate protective potential has been observed. For PLA NPs, CpG-ODN, and PBS groups, lesions progressed rapidly to severe necrosis from 3 to 8 weeks post-infection. In contrast, the footpads of mice vaccinated with PLA-H2B were free of necrosis at the end of the experiment (week 8) and were 2-fold thinner than those of the PBS group (*p* < 0.001). Compared with the CpG-ODN group, the results were significantly similar (Figure 7b).

### 2.8. Evaluating the Parasite Load in Mice

**Figure 8:**
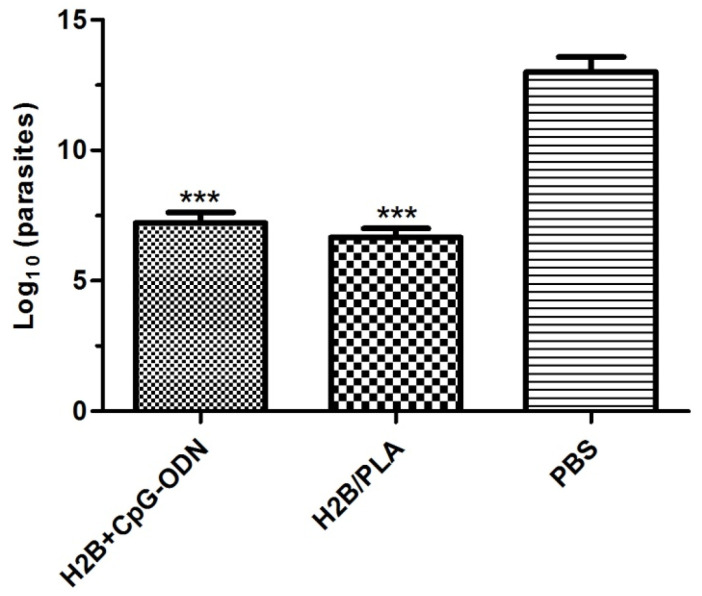
Parasite load in infected footpads. BALB/c mice vaccinated with H2B/PLA and H2B + CPG were challenged with *L. major* promastigotes, respectively. Eight weeks post-infection, footpads were collected. Parasites at the lesion site were counted by limiting dilutions. The results were compared with those obtained from PBS group. (***) *p* < 0.001 between PBS and protein-received group.

Parasites disseminated in infected footpads were quantified using a limiting dilution technique. As expected, mice immunized with PLA (H2B/PLA) and CpG (H2B + CpG) induced good protection against parasites, with 2–3 log fewer parasites than with the PBS group (*p* < 0.001). Unvaccinated mice had the highest parasite load (Figure 8). The results correlated with the lesion thickness observed in respective groups. It suggests that only protein formulated with PLA nanoparticles or CpG-ODN can protect animals against parasite spread. 

## 3. Discussion

A significant public health issue is the absence of effective treatments or preventive vaccinations for several diseases brought on by intracellular infections, such as cutaneous leishmaniasis. Strong cellular responses are needed in the vaccine formulations for these illnesses [34]. Preclinical trials are now being conducted on a variety of potential anti-leishmania vaccine candidates, including live genetically altered parasites, live attenuated parasites, recombinant proteins and DNA vaccines, and vaccinations using antigen combinations [16]. Despite the preclinical effectiveness of some vaccine candidates, their use has been restricted due to the toxicity of a living vaccine on the one hand and the minimal antigenic exposure to the antigen-presenting cells (APCs) for a subunit protein vaccine on the other hand [15,35,36]. However, a satisfactory adjuvant in vaccine formulation has yet to be approved [36]. At present, the WHO still recommends the use of alum in combination with the vaccine antigen; this encourages the investigation of new adjuvants because alum is unable to elicit cell-mediated Th1 response [37].

Some of these limitations can be resolved through the use of nanoparticulate delivery systems. Today, the administration of vaccines to organisms using PLA nanoparticles with surface-adsorbed antigens is a promising approach. The polymer is biocompatible, biodegradable, non-toxic, and non-antigenic [38]. Interestingly, PLA-based vaccines improve efficient cellular immune responses (CTL) to a number of infections [25]. Such an immune response seems crucial for the control of Leishmania, an intracellular pathogen [21].

This research aimed to develop a PLA polymer-based nanoparticle vaccine for the immunization of mice infected with *L. major* parasites. We investigated and compared the immune performance of antigen-coated PLA nanoparticles and CpG 7909, a Th1-activated adjuvant. The recombinant H2B protein, expressed in *E. coli* BL21 strain cells, is a chosen candidate for a leishmanial vaccine [31]. It is interesting to note that this protein does not interact negatively with mammalian histones, is easily expressed in the prokaryotic environment, and is not cytotoxic [39]. The first part of the study was to optimize the physico-chemical conditions of the procedure and the formulation of the vaccine since the size, zeta potential and desorption pattern are parameters that affect the particle uptake by cells. For the development of H2B-coated PLA NPs, we prepared well-dispersed nanoparticles by the nanoprecipitation method. We optimized the protocol so that the particles are obtained in a single step without the use of surface-active agents. This is a benefit in vaccine formulation that overcomes the inherent toxicity of excipients. Many studies demonstrated the emergence of skin toxicity when using surfactants in nano-delivery systems [40]. The PLA-NPs had a mean diameter of 287 nm and had a quite narrow size distribution with a polydispersity index of 0.14. Neither deposits nor agglomerates were noticed in the PLA dispersions. A zeta potential of −45 mV was high enough (in absolute value) to afford colloidal stability through electrostatic repulsion. The carboxyl groups at the particle surface outset the negative global charge. The H2B adsorption on the particle surface resulted in the inversion of the zeta potential from −45 mV to +30 mV. The carboxyl groups were neutralized by protonated amines within the protein backbone. Obviously, histones are classified as proteins with a lightweight structure and a strong basic polycation character [41]. Thus, the H2B adsorption on NPs occurred by electrostatic interactions. The positive global charge of H2B/PLA formulation could optimize cell membrane interactions. This is a crucial parameter of antigen-presenting cells [42]. Several researchers have explored the cell uptake of positively charged particles. Among them, Liu Z. et al. showed that cationic stearylamine lipid-polymer hybrid NPs (LPNPs) of ~200 nm increased the efficacy of AmB through macrophage uptake [23]. As confirmed elsewhere, the sodium alginate-glycol chitosan stearate NPs (AmB-SA-GCS-NP) showed the highest macrophagic uptake in J774.1 macrophages and the rapid in vivo localization tissues of the liver, spleen, lung, and kidney [43]. Many studies have reported that particle size modulates the assimilation of particles into cells. Among them is one concerning PLGA NPs that exhibited low toxicity and efficient uptake by APC in vitro and in vivo when particles were about 300 nm in average size [44].

Finally, the release pattern of H2B/PLA was analyzed at predetermined time intervals for 30 days. The recombinant H2B started desorption on day 7, and the total amount of 30% was reached 3 weeks later (the 30th day).

Weak desorption at pH = 7.4 could be interpreted as a more favorable interaction between protein and polymer than protein and release medium. The progressive H2B release promotes a deposit effect. For vaccination purposes, this can significantly reduce booster doses.

Secondly, we evaluated the vaccine potency of the formulation on the BALB/c mice because they are susceptible to infection with *L. major*, and if allowed to run its course, subcutaneous injection of parasites leads to uncontrolled lesion growth and eventual death [33]. The challenge tests were performed using the standard parasite strain, MHOM/TN/94/GLC94 (GLC94), isolated from a human lesion of cutaneous leishmaniasis (CL). This isolate belongs to the species of *L. major*, zymodeme MON25, and is the most virulent strain [45]. BALB/c mice were subcutaneously immunized two times with the following vaccine formulations: H2B alone, H2B/PLA-NPs and H2B/CpG-ODN7909. We recorded an increase in the average antibody level against H2B-based adjuvant formulations in contrast to groups that were exposed to free antigens. The humoral response intensity (IgG titers) was substantially similar between both formulations and, therefore, immunogenic in a specific way to adsorbed antigens. These results agree with previous work on PLA NPs/Nod ligand formulations, which induced an increase of up to 100-fold in antibody responses against Gag p24 HIV-1 antigen in comparison to alum [46]. As previously described using PLA as an adjuvant, the p24/PLA vaccine induced high antibody titers with a strong CTL response in mice, rabbits and macaque [25]. As for other infectious diseases, the pattern of the IgG subclass has been shown to play a role in the course of *Leishmania* infection. Mice, similarly to humans, show four different classes of IgGs, named IgG1, IgG2a, IgG2b and IgG3, which functionally correspond to human IgG1, IgG2, IgG4 and IgG3, respectively. In general, it is possible to conclude that in mice and humans, IgG1 (as well as IgG4 in humans) is associated with a Th2 profile, and the other subclasses are mainly associated with a Th1 profile [47]. The evaluation of the IgG2/IgG1 ratio allowed us to predict the type of immune response induced by both adjuvants. The PLA/H2B formulation tends towards a Th1-activated response due to the positive IgG2/IgG1 ratio of antibodies against H2B. Clearly, the CpG7909 preferentially activated cellular immunity, as confirmed by the prevalence of IgG2, by directing the differentiation of LB towards the secretion of specific IgG2a antibodies. Additionally, for LCL-infected individuals, PBMC proliferation and IFNγ levels increased significantly in the presence of a recombinant H2B [32]. The prevalence of the cellular response with H2B/PLA could be explained based on preliminary experiments suggesting that the H2B protein has the ability to induce a specific Th1 response in BALB/c mice [31]. When BALB/c mice were vaccinated with the H2B protein alone or in the presence of CpG adjuvant (two injections), they were able, one month after the last immunization, to produce large amounts of IFNγ when their lymph nodes (injection sites) were re-stimulated in vitro with H2B protein. Furthermore, the amounts of IL-10 and IL-4 were very low or not detectable. Such a Th1-polarized response is curative, while the Th2 response exacerbates or is ineffective in controlling the disease [48,49,50].

The challenge assay demonstrated that animals do not develop ulcerating lesions until week 8 post infection. Both groups (PLA particles and CpG-ODN) have the lowest lesion thickness. This suggested that H2B/PLA formulation exhibited a protection immune potency. Consequently, the parasite dissemination has been slowed down or even interrupted in vaccinated animals, while control groups lead to uncontrolled lesion growth and eventual death. Despite the effectiveness of CpG-ODN in triggering a cytotoxic immune response, its use is still restricted since the clearance is rapid. A study of its pharmacokinetics and biodistribution characteristics demonstrated its rapid renal clearance when intravenously injected into animals [51].

A previous study from Khatik et al. (2014) showed that PS–coated gelatin NPs with encapsulated amphotericin B reduced parasite burden in Wistar rats (85% vs. 50%) when compared to standard AmB [52]. For application against CL, AmB nano-encapsulation in PLGA/dimercaptosuccinic acid (DMSA) NPs (nano-DMS-AmB) was developed and tested against C57BL/6 mice infected with *L. amazonensis*. The nano DMS-AmB showed a greater reduction in the number of parasites than standard D-AmB [53]. Nanoliposomes used as the nanocarriers for soluble *Leishmania* antigens (SLA) showed parasite clearance in the footpad and spleen of a mouse model injected with this formulation. Liposomal formulations of ChimeraT (a combination of 3 leishmanial proteins), when delivered subcutaneously, protected mice against *L. infantum* infection by reducing the parasite load in the spleen, liver, bone marrow, and lymph nodes [54].

In the context of leishmanial nano-vaccines, Katebi A. and collaborators (2021) loaded PLGA NPs with SLA and TLR receptor agonists PAM3CSK4 and R848 and incubated them in vitro with immune cell lines. The results showed a marked increment in the macrophages’ phagocytic potential accompanied by a significant reduction in pro-inflammatory cytokines against *L. major* parasites. However, this immunogenic candidate should be tested against a *Leishmania*-infected animal model to determine the in vivo parasitic and disease reduction potential [55,56].

## 4. Materials and Methods

### 4.1. Chemicals and Reagents

Phosphate-buffered saline tablets and acetone were obtained from Sigma-Aldrich Co. (St. Louis, MO, USA). A Low Molecular Weight Calibration Kit for SDS Electrophoresis (Code: 17-0446-01) was purchased from GE Healthcare. An s29 Gx 0.5 syringe (insulin) was purchased from Terumo. The adjuvant CpG-ODN (7698 g/mol) (ODN 7909), a human TLR9 ligand, was purchased from InvivoGen. Poly (D,L-lactide) (PLA Mw = 16,000 g/mol, Mw/Mn = 1.6) was purchased from Corbion Purac Biomaterials (4206 AC, The Netherlands). Imidazole reagent was obtained from GE Healthcare-Application Note 28-4067-41 AA. A QuantiPro BCA Assay Kit was obtained from Sigma-Aldrich Co. (St. Louis, MO, USA)). Schneider’s Drosophila medium was obtained from Gibco-BRL, Paisley, Scotland.

### 4.2. Parasites

The standard strain of the *Leishmania major* parasite is MHOM/TN/94/GLC94 (GLC94), which was isolated in a human lesion of zoonotic cutaneous leishmaniasis (ZCL). The most virulent strain of a parasite is the *L. major* zymodeme MON25 (*L. major*-MON25) [31,32,33]. Promastigotes were collected in a logarithmic-phase culture, counted in a fixed volume, and then incubated once more at 26 °C, as previously mentioned. After five days, the stationary phase was attained, and the parasite concentration value was equal to 4 × 10^7^ parasites/milliliter. Then, using the density gradient centrifugation method, meta-cyclic promastigotes were isolated, cleaned, and administered to BALB/c mice for inoculation (2 × 10^6^).

### 4.3. Mice

Female BALB/c mice (6–8 weeks old) were obtained from The Institut Pasteur de Tunis “http://www.Pasteur.tn (accessed on 12 June 2013)”. The experimental protocol (registry number: 2015/13/I/LR11IPT08 V1) was approved by the Institutional Ethical Bio-Medical Committee of the Institut Pasteur de Tunis IPT, and the US registry number is IRB00005445, FWA00010074).

### 4.4. Preparation of PLA Nanoparticles

Surfactant-free PLA nanoparticles (PLA-NPs) were prepared by the nanoprecipitation method as previously described [57]. Briefly, 0.2 g of polymer was dissolved in 10 mL of acetone and added dropwise to 30 mL of milliQ under moderate stirring water without using a surfactant. The organic solvent and a part of the water were removed by evaporation under reduced pressure. The final NP concentration after the preparation was determined by measuring the solid content after heating the NP dispersion (known volume) to a constant weight in an oven at 70 °C for 24 h. The size distribution was determined by dynamic light scattering technique (DLS) at 25 °C using a Zeta-Sizer Nano ZS (Malvern Instruments, Malvern, UK). Zeta potential was measured by a laser Doppler with the same instrument using diluted colloidal dispersions in 1 mM NaCl.

### 4.5. Expression and Purification of Recombinant Histone H2B

The BL21 *Escherichia coli* (*E. coli*) strain containing the recombinant plasmid pET-H2B was kindly provided by Dr. Chenik.

Purification and expression of the H2B protein were carried out as previously described [31]. Luria broth (LB) medium supplemented with ampicillin (100 µg/mL) and 0.1% glucose was used to grow *E. coli* BL21 pLysS DE3-pET-H2B in shaking flasks until the absorbance at 600 nm reached a value between 0.6 and 0.9. After that, IPTG (1 mM) was used to stimulate protein expression for a minimum of 4 h at 37 °C. Cells were pelleted before the periplasmic proteins were osmotically shocked out and placed into a Ni-NTA column. The histidine-tagged (His-tagged) proteins were then eluted using a linear gradient of imidazole from 0.05 to 0.5 M after being cleaned with PBS. Membrane dialysis was used to desalt fractions. SDS-PAGE was used to evaluate purified proteins, and the BCA assay kit (Pierce, Rockford, IL, USA) instructions were used to calculate protein quantities.

### 4.6. Synthesis of H2B/PLA, a Nanovaccine Formulation

In this study, a nanovaccine formulation was synthesized by the adsorption of H2B onto PLA nanoparticles, as previously described [57]. PLA-NPs were diluted at a concentration of 1.2 mg/mL in Milli-Q water. Then, H2B solutions were prepared at different concentrations (0–100 µg/mL) in NaCl (10 mM). One volume of NPs was added to one volume of protein solution to obtain the H2B/PLA formulation. The adsorption medium has a fixed concentration of both NPs (0.6 mg/mL) and protein, which ranged from 0 to 50 µg/mL (corresponding to an H2B/PLA weight ratio from 0 to 8.3%). Protein and particle dispersions were incubated for 15 min at room temperature with gentle stirring. The samples were centrifuged for 20 min at 5000× *g*. Separate tubes were used to collect each supernatant. Each H2B/PLA pellet was cleaned further by centrifugation/redispersion (5000× *g* for 20 min at 25 °C). The pellets were then resuspended in saline solution (NaCl 10 mM). In accordance with the manufacturer’s recommendations, the QuantiPro BCA assay Kit (Sigma, Germany) was used to quantify the concentration of non-adsorbed H2B. A Zeta-sizer Nano ZS-based DLS technique was used to measure parameters such as the particle size, polydispersity indices and zeta potentials of the H2B/PLA formulations (Malvern Instruments, Malvern, UK).

### 4.7. In Vitro Release Study

To study the desorption profile of H2B, the H2B/PLA pellet (which was used at a 5% *w*/*w* ratio) was resuspended in PBS (pH 7.4) and incubated on a shaker at 37 °C. At set times (12 h, 1, 2, 3, 7, 14, 21 and 30 days), samples (in triplicate) were taken and centrifuged at 5000× *g* for 20 min. Using the QuantiPro BCA assay Kit, the concentration of protein released in supernatants was quantified.

### 4.8. In Vivo Assay

#### 4.8.1. Immunization of BALB/c Mice

In vivo studies have been performed in BALB/c mice, which are the mouse models used for preclinical evaluation of *Leishmania* vaccine candidates [33]. Forty-eight females, between 6 to 8 weeks old, were used in the experiments. The animals were divided into 6 groups, including 8 mice each. They were then administered subcutaneously twice on days 1 and 14 with the following vaccine formulations: H2B alone (25 µg/injection), H2B (25 µg) plus CpG (20 µg), H2B/PLA-NPs (25 µg/0.9 mg), CpG (20 µg), PLA-NPs (0.9 mg) and PBS. In each vaccination, 200 µl of the preparation diluted in PBS was injected into the animals. On day 30, blood samples were taken to measure serum antibody levels.

#### 4.8.2. Identifying the IGg1 and IgG2 Isotypes

To evaluate the antibody pattern generated based on the H2B/PLA formulation, the IGg1/IgG2 subtype was examined. Enzyme immunoassay (ELISA) was used to detect specific antibodies generated against H2B formulations (H2B/PLA and H2B + CpG7909) in mouse serum. Briefly, 5 µg/mL of H2B was coated in 100 µL of carbonate-bicarbonate buffer (0.1 M) and incubated overnight at 4 °C in 96-well high-binding plates (Nunc). PBS-T20 buffer was used three times to wash the plates. PBS-T20 solution, supplemented with low-fat milk (2%), was used to block the plates at 37 °C for 1 h. After the wash steps, sera were added at a dilution of 1/1000 and incubated for 2 h at 37 °C.

#### 4.8.3. Parasite Infection and Development of Lesions

The purpose of the treatment was to demonstrate an adjuvant effect of H2B/PLA against an experimental *L. major* infection. A minimum of 6 weeks was required between vaccination and infection. Six weeks after receiving the booster dose, animals in each group were challenged with infectious *L. major* promastigotes (GLC94) in order to observe and evaluate the efficacies of both the H2B + CpG and H2B/PLA-NPs vaccine formulations. For this, mice were subcutaneously injected with an infective parasite load (2 × 10^6^) of *L. major* promastigotes suspended in 50 µL of PBS in the right footpad. Within eight weeks, the swelling’s growth was tracked once a week using a dial-gauge caliper (Mitutoyo, Japan). Lesion values (in mm) were calculated by subtracting the thickness of the infected footpad from the thickness of the contralateral footpad that was not infected.

#### 4.8.4. Determining the Parasite Load

Parasite load was quantified by a limiting-dilution technique adapted from the work of Laskay et al. [58]. The infected pad was cut (3 groups, *n* = 8) and homogenized before serial 10-fold dilutions were plated in triplicate in 96-well flat-bottom microtiter plates (Nunc, Roskilde, Denmark) containing Schneider’s Drosophila medium (Gibco-BRL, Paisley, Scotland) with the addition of 100 U of penicillin/mL, 100 µg of streptomycin/mL, 2 mM L-glutamine, and 10% heat-inactivated fetal calf serum. Plates were incubated at 26 °C. Live parasites were attested under an inverted microscope. Parasite load is expressed as the average of the log negative of the last dilution in which mobile parasites were detected.

## 5. Statistical Analysis

Statistical analysis was performed using GraphPad Prism software 5.1 (GraphPad Software 2365 Northside Dr. Suite 560 San Diego, CA 92108). The data are expressed as mean ± standard deviation. Statistical significance *p*-values of less than 0.05 are considered statistically significant.

## 6. Conclusions

The successful development of an effective vaccine needs exact data regarding the physicochemical characteristics of the antigen and adjuvant, as well as expertise in their combination to generate a safe, steady, and immunogenic vaccine.

Our study described the immunogenicity and protective capacity of H2B/PLA as a particulate vaccine in an anti-leishmanial therapeutic setting. The outcomes clearly demonstrate that the PLA nanoparticles serve as an adjuvant for the recombinant protein H2B since BALB/c mice were protected against experimental infection by the *L. major* parasite, a standard virulent strain GLC94. In contrast, mice immunized with a soluble protein were unable to stop the parasite’s spread, which led the paw to completely ulcerate. The H2B/PLA’s added value refers to its colloidal stability, simple manufacturing process, and established adjuvant capacity.

It will be interesting to extend these data by incorporating immunostimulatory molecules (such as imiquimod, 3 M 052) in the NP PLA core to increase its protective effect.

## Figures and Tables

**Table 1:** Main characteristics of H2B/PLA-NPs selected for animal experiments.

Adsorption Capacity % *w*/*w*	Zeta Potential mV	DLS Size nm
2.8 (±0.24)	30.95 (±0.78)	340.8 (±52)

## Data Availability

Data available from the corresponding author.
